# Mapping Retinal Gene Expression to Cell Classes: Insights into Retinal Circadian Rhythms

**DOI:** 10.3390/clockssleep8040060

**Published:** 2026-09-25

**Authors:** Julia Buckland, Michael J. Gilhooley, Mark W. Hankins, Ma’ayan Semo, Stuart N. Peirson

**Affiliations:** 1 Sleep and Circadian Neuroscience Institute, Kavli Institute for Nanoscience Discovery, Nuffield Department of Clinical Neurosciences, University of Oxford, South Parks Road, Oxford OX1 3QU, UK; 2 Neuro-Ophthalmology and Adult Strabismus, Stein Eye Institute & Doheny Eye Institute, University of California Los Angeles, Los Angeles, CA 90095, USA

**Keywords:** circadian, retina, photoreceptors, transcriptomics, bioinformatics, vision, single-cell RNA sequencing, cell-class specificity, retinal cell mapping

## Abstract

Many retinal processes are regulated by circadian rhythms. Previous studies have identified genes with rhythmic retinal expression over a 24 h cycle, but the cell class specificity of many of these genes remains unknown. Here we present a mathematical model, based on existing human retinal single-cell RNA sequencing (scRNA-seq) data and validated using known retinal markers, which assigns cell classes to genes showing uniquely enriched expression in that class. This model assigns retinal cell class specificity to over 10,000 human genes and provides potential novel candidates for retinal cell markers. Additionally, the model enables transcriptomic datasets to be investigated through cell class enrichment analysis. We apply this model to circadian-expressed genes from published mouse ocular transcriptomic data. Specific cell classes were assigned to over 35% of these genes, many of which have little to no previous literature denoting their retinal localisation and role. We also demonstrate significant enrichment of photoreceptor-associated transcripts, many of which are associated with phototransduction and outer segment turnover. The methods and data presented here provide a resource for researchers interested in the cell class specific expression of retinal genes and provide new insight into the cellular basis of retinal circadian rhythms.

## 1. Introduction

To anticipate the 24 h cycle of night and day, many processes across different cells and tissues are under circadian control. As the tissue which detects and transduces spatial and temporal variations in light intensity for vision, the vertebrate retina undergoes dramatic changes over the 24 h cycle allowing adaptation to light levels throughout the day [1,2,3]. The retina also relays information to the suprachiasmatic nucleus, entraining internal circadian rhythms to the external light/dark cycle [4]. This allows entrainment of rest/activity patterns, as well as mediating a wide range of non-visual responses to light such as pupil constriction, melatonin and cortisol synthesis, sleep, cognition and mood [5].

The retina contains autonomous molecular clocks dependent on a transcriptional-translational feedback loop (TTFL). The core clock proteins CLOCK and BMAL1 form a heterodimeric transcription factor, interacting with the E-Box element to upregulate transcription of *PER1* and *PER2* and *CRY1* and *CRY2*. The resulting PER and CRY proteins dimerise and inhibit BMAL1/CLOCK activity. An accessory loop is formed as BMAL1/CLOCK activates the transcription of RORα and REV-ERBα, which respectively promote or inhibit *BMAL1* transcription, driving rhythmicity of its activity [6]. In addition, E-boxes regulate the expression of many clock-controlled genes which are thought to be involved in tissue-specific physiology. Many retinal processes are under the direct or indirect control of the TTFL, including dopamine and melatonin synthesis [7,8], photoreceptor outer segment disc shedding [9], ERG responses [10,11] and photoreceptor coupling [12,13,14].

Circadian gene expression can be analysed using whole tissue (bulk) methods such as microarrays or bulk RNA sequencing (RNA-seq), or by sequencing of individual cells by single cell RNA sequencing (scRNA-seq). Though scRNA-seq allows for localisation of gene expression at the level of individual cells, there are technical challenges when using scRNA-seq data to compare the retinal transcriptome across multiple time points. Conversely, bulk retinal RNA sequencing cannot report cell class specificity, although this is both cheaper and more convenient at multiple time-points. One approach to addressing this problem is to use whole tissue methods to identify rhythmic genes and then use statistical techniques to mathematically separate predicted individual cell class expression profiles from the bulk RNA-seq expression data. Many such approaches exist, which are together referred to as deconvolution [15]. The existence of discrete retinal cell classes with unique transcriptomic signatures would benefit such deconvolution approaches, compared to many tissues where gene expression may vary subtly between cell class, necessitating probabilistic models [16]. These deconvolution approaches may, however, be inappropriate within the retina, as astrocytes, microglia, retinal ganglion cells (RGCs) and horizontal cells each constitute 1% or less of the cells in the retina [17,18], which is below the minimum proportional threshold of the majority of these methods [19]. Also, statistical deconvolution usually requires accurate knowledge of cell class proportions, which is rarely available for historical datasets. Whilst proportions can be estimated from other datasets, these may be inaccurate, as proportions of different cell classes, such as RGCs, vary between individuals [20], as well as different strains of the same species [21]. As such, existing statistical deconvolution methods are not often viable for analysing existing retinal bulk RNA-seq datasets, limiting their application for establishing retinal cell class specificity for genes associated with circadian function.

As different retinal cell classes have remarkably specific and well-characterised functions compared to other tissues, cell class localisation of gene expression is often a key step in understanding the results of retinal transcriptomic studies. Here we develop, validate and apply a minimalistic model to assign specific cell class to genes in the retina (Supplementary Data S1). We used published human retinal scRNA-seq data [22], clustered to ten different cell classes: rods, cones, horizontal cells, bipolar cells, amacrine cells, RGCs, microglia, astrocytes, Müller cells and ‘others’. This data was chosen due to its sequence depth of 40,232 reads per cell across 23,000 cells, which gave expression data for each human gene and enabled the major cell classes of the retina to be identified. The model reports the expression of each gene to be specific to a cell class if its expression in that cell class meets a criterion value for expression level and expression specificity. To validate this approach, we used a data set of 81 genes identified from the literature that are known to be cell class specific in the retina across mammalian species. As well as assigning cell class, this enables enrichment of genes specific to retinal cell classes to be determined within any list of genes. This model provides a tool for the retinal research community, providing cell class specificity in retinal transcriptomic data from whole tissue analysis. We go on to apply this method to published retinal circadian data consisting of 206 rhythmic retinal transcripts identified through bulk RNA-seq, assigning cell class specificity to 84 of these (41%). These data provide new insight into the cellular basis of retinal circadian rhythms, highlighting a large number of photoreceptor specific transcripts under circadian control.

## 2. Results

### 2.1. Literature Survey

A workflow diagram for the development of the retinal cell mapping model is shown in Figure 1. Based on the published literature, we identified 83 genes reported to be specific to a single retinal cell class in vivo. For the purposes of this study these did not need to be ubiquitously expressed in all cells of that class. Of these, two (*POU4F3*, *OPN4*) were excluded due to the absence of detectable expression in the source dataset. A total of 81 genes with strong *in vivo* evidence of retinal cell class specific expression across mammalian species were subsequently used, with evidence including immunohistochemistry, immunofluorescence and in situ hybridisation. These marker genes are listed in Table A1 (Appendix A), as well as the species in which each gene was experimentally validated. Markers were found for the following primary retinal cell classes: rods (14 genes), cones (12 cells), horizontal cells (4 genes), bipolar cells (14 genes), amacrine cells (8 genes), retinal ganglion cells (11 genes), Müller cells (6 genes), astrocytes (4 genes) and microglia (8 genes).

### 2.2. Characterisation of Unassigned Clusters

In the dataset used, a subset of cells was classified as ‘others’ [22]. This category, consisting of two clusters (C5 and C14), was investigated in more detail to determine if this data could be used to identify other cell classes of interest. Increased expression of rod and Müller cell markers in C5, and of rod and cone markers in C14, was demonstrated. Mean expression in these clusters of the marker genes for each of the defined cell classes was compared. This was found to be significantly enriched for rod markers in C5 and for rod and cone markers in C14 above other cell classes (Supplementary Figure S1a). The ‘others’ category showed moderate but inconsistent expression of retinal pigment epithelium (RPE) markers (Table A1, Supplementary Figure S1b), and no appreciable expression of the endothelial markers CD31 and CD146 (Supplementary Figure S1c), so likely does not uniquely represent either of these cell classes. As the ‘Others’ cluster expresses assorted markers of multiple retinal cell classes, it most likely represents assorted debris from rods, cones and possibly Müller cells and RPE. As such, this category was excluded from further analysis. Percentage expression values for each gene in each cell class were recalculated accordingly.

### 2.3. Retinal Cell Markers

Based upon the percentage of total retinal expression, the cell class-specific markers identified from the literature were found to be highly expressed in the expected cell class (Figure 2). Some variability was observed in amacrine cell markers and to a lesser extent in retinal ganglion cell markers.

### 2.4. Model Development and Validation

Defining a gene as cell class specific based upon exclusive expression in one retinal cell class is of limited value. Expression of a gene may be sufficiently enriched in one particular cell class to be considered specific to that cell class, whilst still being expressed at lower levels across other cell classes. As such, to improve our criteria for assigning genes as cell class specific, we used a simple two parameter model. To be cell class specific, a gene should be expressed at a high level in a specific cell class (enrichment), and this expression should be largely restricted to just that cell class (specificity). We defined enrichment as the minimum percentage of total retinal expression which must be found in one cell class. Specificity was defined as the maximum percentage of the expression level in that cell class which is permitted in a different cell class. The enrichment and specificity values at which each marker failed to be identified as cell class specific was determined for each marker gene. The optimum parameters identified were 30% for enrichment, and 65% for specificity (Figure 3a). In practice, this means that expression in one cell class accounts for at least 30% of the total retinal expression, and this cell class has an expression that is at least 1.54 times more than the next highest expressing cell class. This represents empirical selection of the points beyond which false positives (genes wrongly assigned as cell-specific) and false negatives (genes which are not correctly assigned as cell-specific) start to significantly increase, and reflects a balance between sensitivity and specificity.

Based upon these values, 72 of the 81 marker genes (89%) were reported as cell class specific (Figure 3b). Of the nine markers that failed, six were most highly expressed in a cell class different to the one they were reported to be a marker for (*LHX9*, *SLC6A1*, *SLC6A9*, *MAP1A*, *RBPMS*, *NDRG2*), whilst three (*GNAO1*, *GAD1*, *GLUL*) also showed high expression in a second cell class and thus failed at the specificity parameter (Supplementary Figure S2). Applying these validated parameters to the 21,989 human genes in the source dataset, 10,748 were assigned to specific retinal cell classes. Considering only the 14,596 genes in the dataset with 1:1 human to mouse homology, 6007 mouse genes were assigned to specific retinal cell classes (Figure 3c, Supplementary Data S2).

### 2.5. Addition of ‘Photoreceptors’ Category

As highly specialised sensory receptors, many processes such as phototransduction and outer disc shedding are common and unique to both rods and cones. Therefore, the ‘photoreceptors’ category was added, to identify genes highly expressed in both rods and cones. This allows identification of genes relevant to retinal function which would not reach the thresholds to be labelled as cell-type specific in rods or cones alone, but would be specific to photoreceptors if considered together as a group.

For each gene in this category, the cell classes accounting for the highest and the second highest percentage of retinal expression must be the two photoreceptor cell classes. Using eleven experimentally validated photoreceptor markers identified in the original literature search (Table A1, Supplementary Figure S3a), the enrichment and specificity parameters for this category were revalidated. The enrichment parameter here refers to a minimum percentage expression which both rods and cones must surpass, and the specificity parameter to the maximum percentage of expression permitted in another cell class (non-rod, non-cone). The values for each of these at which each marker was no longer designated as photoreceptor-specific were determined (Supplementary Figure S3b). Parameters were set at 25% for the enrichment parameter, and 55% for the specificity parameter (Supplementary Figure S3c). Genes were only assigned to the ‘photoreceptors’ category if they did not meet the parameters for being uniquely rod- or cone-specific; three photoreceptor marker genes (*ELOVL4*, *FSCN2*, *RCVRN*) were denoted as rod-specific. At these parameters, 210 human genes and 126 mouse genes with 1:1 human homology were assigned to this category (Supplementary Data S2).

### 2.6. Application to Retinal Circadian Data

The validated retinal cell mapping model was first applied to rhythmic genes in constant darkness (DD) in the mouse eye [11], following exclusion of duplicate genes. Of the 234 unique rhythmic genes, 21 had no 1:1 human ortholog, whilst the human orthologs of 7 more were not expressed in the source scRNA-seq dataset. A total of 84 of the remaining 206 genes (40.8%) were assigned specific cell classes (Figure 4a, Supplementary Table S1). Cell class enrichment analysis found rods, cones and photoreceptors to be significantly enriched amongst the cell class categories assigned to these genes (Figure 4b).

### 2.7. Application to Retinal Diurnal Data

The model was also applied to rhythmic genes in diurnal conditions (LD) from the mouse eye. Of these 832 genes, 93 had no 1:1 human ortholog, whilst the human orthologs of 16 were not expressed in the source dataset. A total of 271 of the remaining 723 genes (37.5%) were assigned specific cell classes (Supplementary Table S2). Again, cell class enrichment analysis found rods, cones and photoreceptors to be significantly enriched amongst the cell classes assigned to these genes (Figure 4c). The rod-, cone- and photoreceptor-specific genes from the LD and DD datasets demonstrated near-complete overlap, as the DD genes were a subset of the LD genes, with only two exceptions (*Best2*, *Slc25a20*) in cones and one (*Cldn7*) in photoreceptors (Figure 4d).

### 2.8. Disease Relevance

Cell class specific rhythmic genes were queried against the RetNet database of 348 human genes causing monogenic retinopathy [23]. A total of 14 cell class specific circadian genes (16.7%) in constant darkness and 32 (11.8%) in diurnal conditions were associated with retinopathy (Supplementary Table S3). Notably, this included over 30% of rod and photoreceptor-specific circadian genes in both DD and LD, and over 20% of cone-specific circadian genes in LD (Figure 5).

## 3. Discussion

Here we present an approach to retinal cell mapping for transcriptomic data, validated using established retinal markers for key cell classes. This enables identification of genes displaying cell class specific expression in the retina based upon existing scRNA-seq data [22]. We go on to apply this model to published circadian and diurnal gene expression data from the mouse eye [11], showing an enrichment of photoreceptor specific transcripts. These findings suggest that photoreceptors account for a large proportion of circadian transcriptomic changes in the retina. These photoreceptor circadian variations may underlie anticipation of changing light levels across the day/night cycle, including rod-specific (*Sag*, *Pde6b*, *Pde6g*, *Rcvrn*), cone-specific (*Arr3*, *Guca1b*) and photoreceptor-specific (*Guca1a*) phototransduction components. As it is well known that many genes associated with inherited retinal diseases directly affect photoreceptor function, a high proportion of our photoreceptor-specific circadian genes were also linked to monogenic retinopathies. These data suggest a role for circadian rhythms in retinal disease, supported by findings that disrupted retinal circadian rhythms accelerate retinal degeneration in mouse models [24].

Many rhythmic rod-, cone- and photoreceptor-enriched genes have confirmed or potential roles in outer segment shedding, renewal and trafficking (Table 1). This is consistent with previously reported enrichment of genes associated with cilial assembly amongst daytime-peaking circadian transcripts in the mouse retina [25], likely representing rod rather than cone outer segment renewal as this occurs maximally during the daytime [26] and rods constitute over 97% of murine photoreceptors [17,18]. The circadian expression of many of these genes has not been independently established beyond transcriptomic analyses, so they warrant further investigation as they may be central to the autonomous circadian regulation of photoreceptor outer disc renewal. It must be noted that any patterns in transcript expression may not necessarily be represented at the level of the protein. Conversely, changes in protein abundance may not be reflected by transcript changes [27]. As such, further immunohistochemical validation of both the rhythmicity of these proteins and the cell class specificity of this rhythmicity would be valuable for understanding their role in circadian retinal function.

Other cell class-specific genes are directly involved with the core circadian machinery, as well as rhythmicity of retinal dopamine and melatonin. One such gene is rod-enriched *Usp2*, one of the few genes reported as rhythmic in mouse DD and LD conditions. Its specificity is surprising considering its ubiquitous nature; *Usp2* is one of only ten genes demonstrated to show rhythmic expression over 12 tissues [44]. USP2 deubiquitinates and stabilises BMAL1 [45] and CRY1 [46], and promotes proper PER1 nuclear localisation [47]. Peak *Usp2* transcript levels are seen at ZT8-12, at the light–dark transition, and *Usp2*^−/−^ mice show greater sensitivity to low irradiance light at that time [45]. This role of *Usp2* in regulating sensitivity to scotopic light at the light-dark transition is consistent with its rod enrichment. Additionally, rod-specific *Hsp90aa1* encodes the α-subunit of the inducible isoform of HSP90, a highly abundant molecular chaperone [48] which stabilises BMAL1. HSP90 inhibition reduces BMAL1 protein and mRNA levels, decreases BMAL1 circadian amplitude and delays its phase [49]. HSP90 is essential for photoreceptor survival [50,51], and side-effects of HSP90 inhibitors include night blindness and delayed dark/light accommodation [52,53], indicating rod dysfunction. This is consistent with rod enrichment of *Hsp90aa1*. HSP90 also stabilises rod phosphodiesterase [54], the subunits of which are expressed in a circadian, rod-specific fashion, as well as MAP1B to allow proper rhodopsin trafficking and outer segment organisation [55]. This additionally implicates HSP90 in facilitating both phototransduction and rod outer segment turnover. Furthermore, cone-enriched *Adcy1* encodes adenylate cyclase, and rhythmicity of *Adcy1* mRNA is dependent on dopamine action at D4 receptors [56] and leads to a peak in photoreceptor cAMP content at night. This stimulates AANAT activity, facilitating circadian melatonin secretion [57]. For further discussion of the potential biological relevance of the rhythmic genes identified as astrocyte-specific in our model please see the Supplementary Discussion.

Analysis of scRNA-seq data showed that expression of a small proportion of the established retinal cell markers differs from previous reports. Two experimentally validated retinal ganglion cell markers, *OPN4* and *POU4F3*, had no detected expression in the source dataset. *OPN4*, melanopsin, is expressed by a subset of retinal ganglion cells known to be intrinsically photosensitive (ipRGCs) [58], which have been shown through scRNA-seq to make up only 12% of RGCs [59]. Though less strikingly subtype-specific, *POU4F3* has additionally been shown to be less ubiquitously expressed across RGC subtypes than many of the other RGC markers identified in our literature search [59]. As RGCs themselves only make up 0.5–1% of retinal cells [17,18], it is likely that the absolute level of *OPN4* and *POU4F3* mRNA is below the threshold of sensitivity to be detected in the source dataset.

Six markers were most highly expressed in a cell class different to the cell for which they were reported to be a marker (*LHX9*, *SLC6A1*, *SLC6A9*, *MAP1A*, *RBPMS*, *NDRG2*). For *LHX9*, *MAP1A* and *NDRG2*, immunohistochemical localisation data could not be found for human retina, so this may reflect either differences in transcript versus protein expression, or interspecies differences in localisation. It is interesting that *RBPMS* expression was not found to be specific to RGCs as it is a well-established immunohistochemical marker for these cells, suggesting that some caution may be needed in utilising *RBPMS* as an RGC marker at the transcriptional level due to its expression in astrocytes. *NDRG2* is one of only four astrocyte-specific markers identified, along with *PAX2*, *PAX8* and *GFAP*. Though GFAP is specific to astrocytes in health, it is upregulated in Müller cells in response to retinal injury [60,61,62,63]. This highlights the need for more robust astrocyte-specific retinal markers, particularly over varying tissue states. Additionally, though *SLC6A1* and *SLC6A9* are reportedly amacrine-specific in human retinal tissue [64,65], both show high RGC expression in the source scRNA-seq dataset. Expression of *SLC6A9*’s protein product Glyt-1 in the ganglion cell layer has been previously attributed to displaced amacrine cells [66]; this may therefore indicate a previously underestimated contribution of RGCs to Glyt-1 expression, or alternatively post-transcriptional modification unique to RGCs may limit translation of *SLC6A9* mRNA in these cells. However, this does not explain the absence of *SLC6A9* expression in amacrine cells in this dataset, contradicting previous studies which demonstrate strong amacrine Glyt-1 immunoreactivity [65,66,67]. The three remaining marker genes not assigned to the correct cell class are *GNAO1*, *GAD1* and *GLUL*, due to insufficient specificity of expression. *GLUL*, encoding glutamine synthetase, is a widely used Müller cell marker validated in many mammalian models [68,69,70,71], yet was also highly expressed in astrocytes. This does not necessarily preclude its use as a Müller cell immunohistochemical marker, as Müller cells and astrocytes are distinguishable by location and morphology. However, this casts doubt upon the use of *GLUL* as a unique Müller cell marker at the transcriptional level, particularly in human tissue.

It is important to discuss the potential limitations inherent in the model we describe. Firstly, this is based on human scRNA-seq data translated to mouse circadian data. This approach was deliberately chosen to facilitate translation between mouse models and human retinal physiology. Whilst the mouse retina is inarguably the primary preclinical model of human retinal function, the retinas of mice and humans have a number of important differences, most notably the absence of a macula in mice. Nevertheless, the mouse retina has rod and cone densities comparable to those of the human peripheral retina, supporting its utility as a model for studying retinal function outside the specialised macular region [72]. More broadly, mouse models have provided a huge number of advances in both our understanding of retinal function and treatment of retinal disease. Mouse and human genetics also differ, although 70–80% of protein encoding genes are identical between species. Non-identical protein coding genes may reflect gene families that have undergone species-specific expansions or reductions, or changes in reading frame. Despite this genetic similarity, differences in regulatory regions may exist, with consequences for transcriptional regulation [73]. In this study, we therefore attempted to limit the potential effect of differing gene expression patterns in the retina between mouse and humans by only considering genes in the model with 1:1 mouse to human orthology.

In addition, rhythmicity of transcript levels does not guarantee rhythmicity of protein function. The converse is also true, and many rhythmic proteins lack corresponding rhythmicity at the transcript level [27,74,75,76]. This may mean that the range of cell class-specific rhythmic retinal proteins may extend beyond those identified in this study due to translational and post-translational control. The source scRNA-seq dataset also may potentially represent a daytime or baseline state, which may not reflect the source of experimental samples. For example, expression of *AANAT*, the rate-limiting enzyme in melatonin synthesis, is undetectable in photoreceptors in the source dataset, where its expression is rhythmic and highest at night [77,78,79]. This model may therefore miss some high-amplitude circadian transcripts which peak at night, so separate characterisation of these may be required. An additional consideration when translating between mouse and human retinal transcriptomic data is that mice are nocturnal whereas humans are predominantly diurnal. Differences in temporal niche may be associated with species-specific differences in the phase or regulation of retinal processes across the light–dark cycle. As rhythmicity in this study was studied exclusively from mouse datasets, these differences should be considered when interpreting their relevance to the human retina.

An awareness of the impact of deviations from the baseline state may be particularly important when applying this model to data from studies of disease states, wherein expression patterns of transcripts and therefore their cell-class specificity may change between different cell classes as a result of disease progression or in response to experimental stimuli. This is supported by the example of *GFAP* described above. Additionally, the scRNA-seq dataset from which this model was derived collated data only from three individuals, with ages ranging from 42 to 80. Given known inter-individual variability in transcriptomic profiles [80], future improvements to this model may include utilising larger scRNA-seq datasets. Finally, we recognise that this work does not investigate enrichment of known subsets within cell classes, with possible examples being the aforementioned RGC subsets, S-cones and L/M cones, ON and OFF bipolar cells. Application of similar mathematical principles in this context could be a potential avenue for future work.

Here we present a minimalistic model for mapping retinal genes to specific cell classes and calculating cell class enrichment in retinal gene datasets. We go on to apply this model to published retinal circadian data demonstrating enrichment of photoreceptor-associated transcripts. Though there is some understanding of the cell class specific function of many identified genes, the function of others remains unknown. However, these may provide useful markers of retinal cell class when studying retinal phenotypes in both mouse and human disease. Future studies may focus on validation of the expression of these genes in their assigned cell class using anatomical methods, to support the in vivo translatability of this in silico work. This approach may also be useful for researchers studying behaviour in transgenic mouse models, where retinal expression is often overlooked. Here, retinal expression may aid in the interpretation of behavioural phenotypes. For example, impaired performance in learning and memory tasks may be influenced by differences in responses to visual cues rather than effects on cognitive processes. Overall, this work serves as a useful starting point for further independent identification and validation of the role of key circadian transcripts and proteins underlying the intrinsic rhythmicity of retinal functions, particularly those whose retinal cell class specificity was previously unknown.

## 4. Materials and Methods

### 4.1. scRNA-Seq Data, Gene Nomenclature and Homology

Relative human retinal scRNA-seq gene expression level data was obtained from Lukowski et al. 2019 [22]. Clusters representing the same cell class were averaged weighted by cluster size, giving relative expression values across 10 cell classes (rods, cones, horizontal cells, bipolar cells, amacrine cells, RGCs, Müller cells, astrocytes, microglia and “others”) for 21,989 genes. From this, percentage expression in each cell class was calculated for each gene. Outdated gene names were replaced with their official symbols from the HUGO Gene Nomenclature Consortium (HUGO). Human and mouse gene homology data was obtained from Mouse Genome Informatics (MGI), and protein-coding genes with 1:1 human to mouse homology were identified following exclusion of pseudogenes. Retinal disease-associated genes were obtained from RetNet [23].

### 4.2. Literature Search for Markers

A workflow diagram for development of the retinal cell mapping model is shown in Figure 1. First, experimentally validated retinal cell-specific markers were identified in a pragmatic search of NCBI PubMed, initially using the phrases ‘retinal cell specific[title]’, ‘retinal cell marker[title]’, ‘retinal marker[title]’ and ‘retinal cell localization[title]’. As well as primary papers providing evidence for cell class specificity directly identified in this search, any other genes implicated as markers were followed up to yield appropriate primary evidence for their specificity.

### 4.3. Analysis of Gene Lists

Once appropriate parameters for assigning cell class specificity were determined and validated using the retinal cell class specific markers identified in the literature search, a simple application was built to report retinal expression patterns and cell class specificity of single genes (Microsoft Excel). This toolbox also outputs lists of genes specific to each cell class from inputted gene datasets, and assesses enrichment of cell classes using one-tailed Fisher’s exact test (Supplementary Data S1).

## Figures and Tables

**Figure 1:**
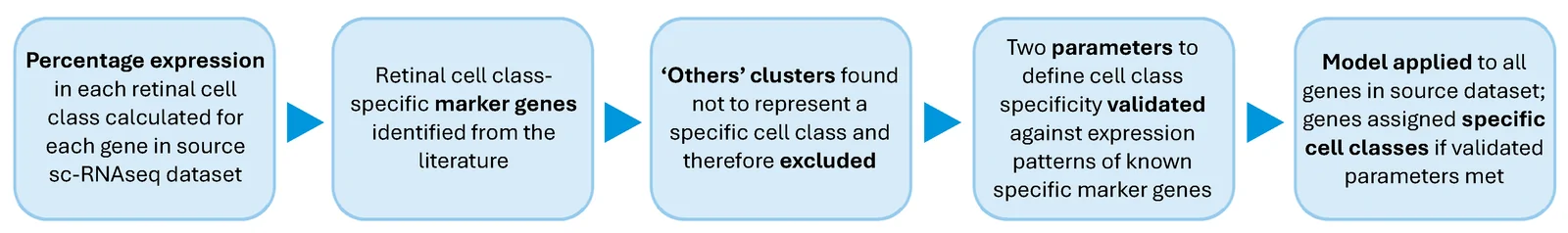
Workflow showing development of the retinal cell mapping model.

**Figure 2:**
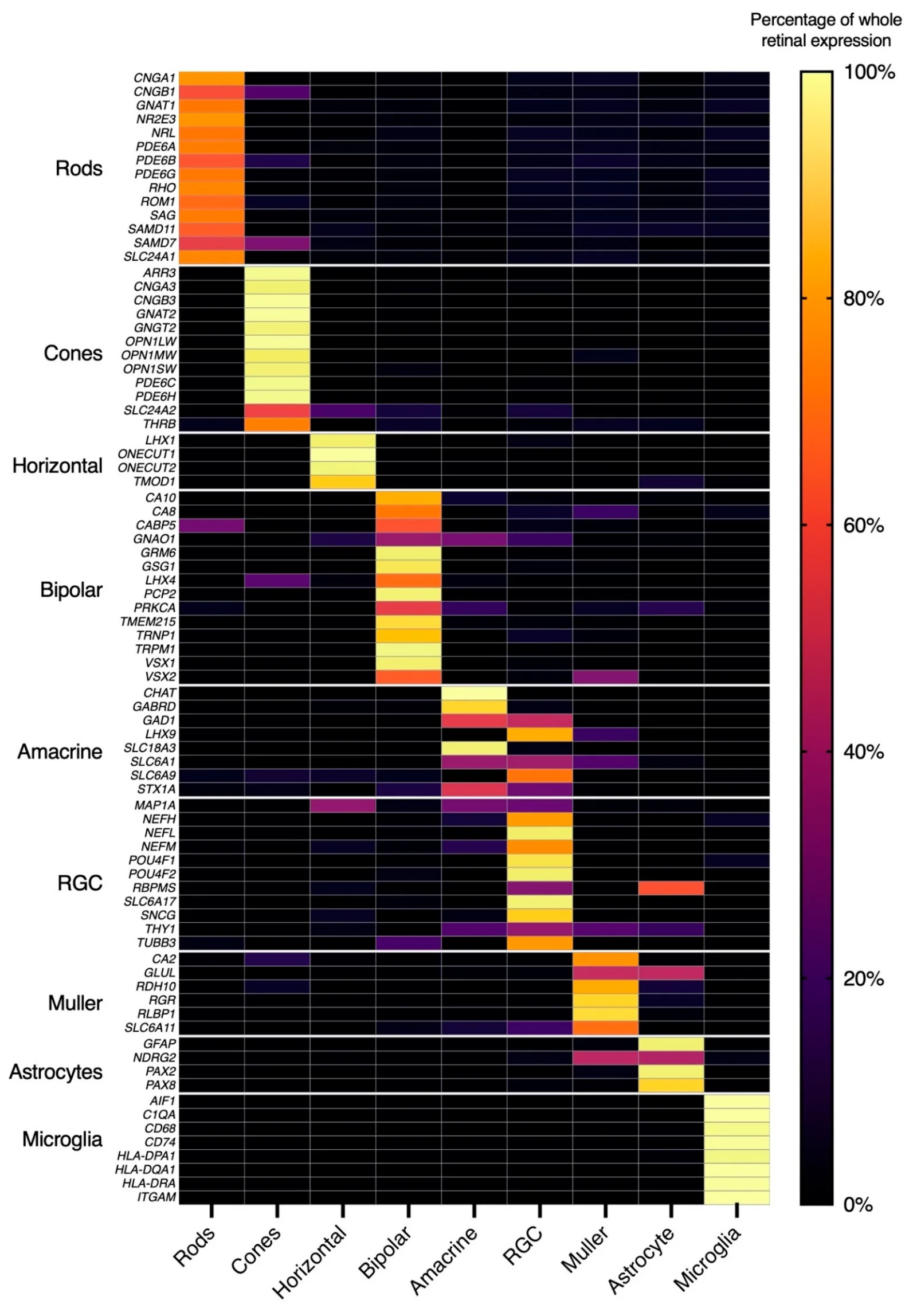
Heatmap displaying percentage of expression of retinal cell marker genes in each retinal cell type. Retinal cell marker genes identified from published literature as described in Section 4. Expression data from Lukowski et al. 2019 [22].

**Figure 3:**
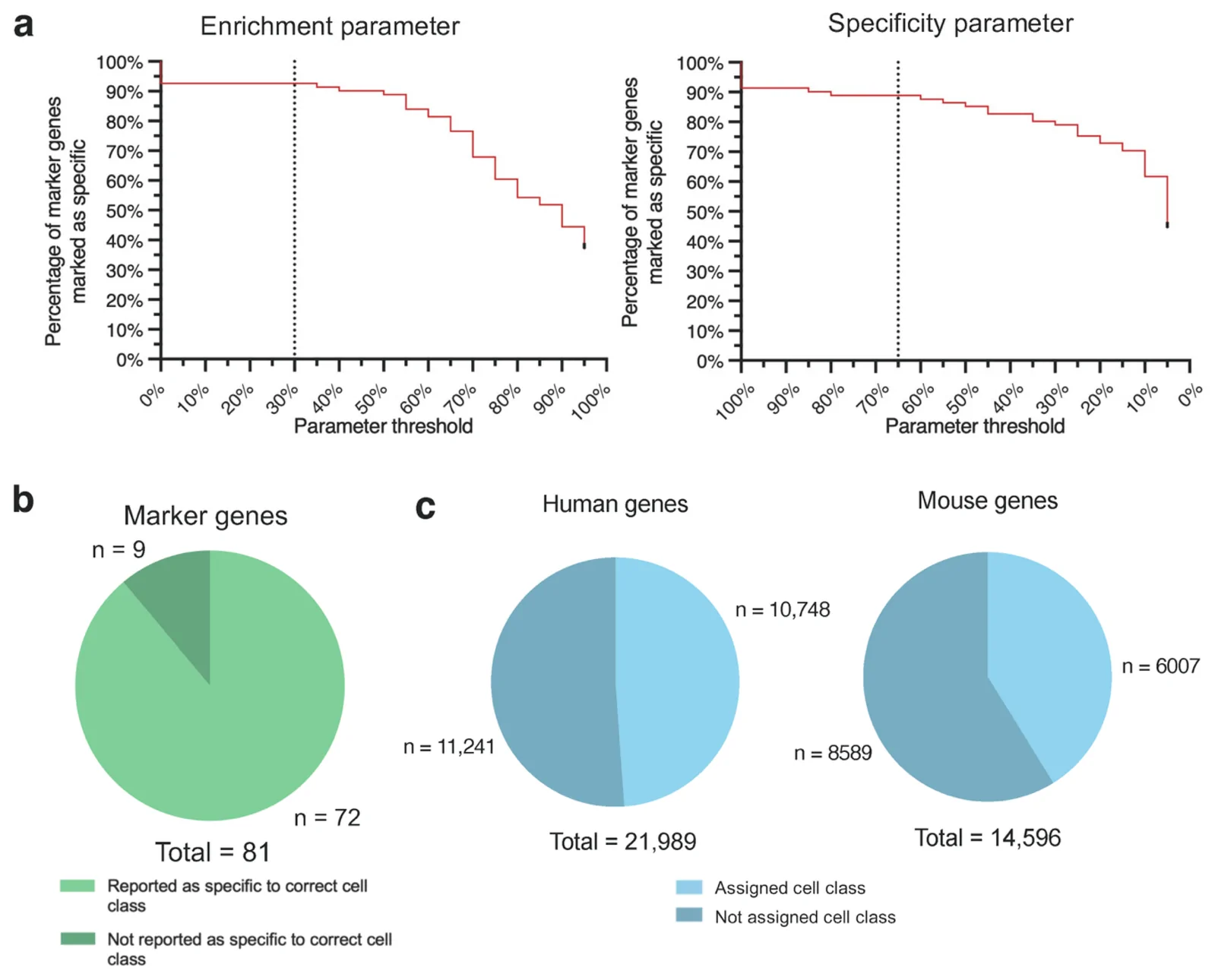
Determination of appropriate model parameters to define cell class specificity. (**a**) Percentage of marker genes reported as cell class specific at each parameter value for the expression parameter and specificity parameter. Finalised parameter values are indicated with a dotted line. (**b**) Proportion of marker genes reported as cell class specific at the finalised parameters. (**c**) Proportion of all human genes, and mouse genes with 1:1 mouse to human homology, reported as cell class specific at the finalised parameters.

**Figure 4:**
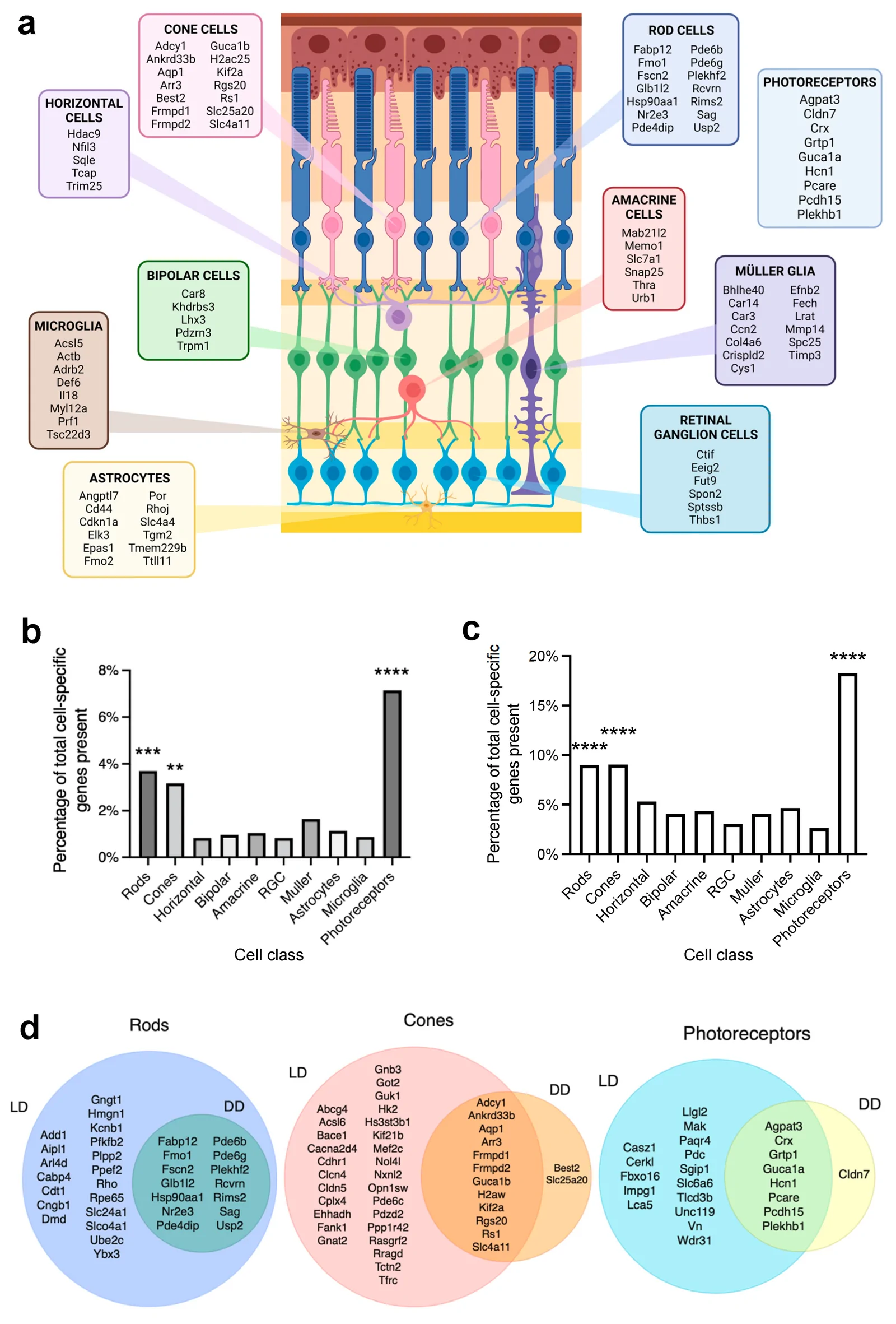
Cell class mapping of circadian genes in the mouse retina. (**a**) Genes with rhythmic expression in the mouse eye in constant darkness which were assigned specific retinal cell classes. Image created in BioRender. (**b**) Statistically significantly enriched cell classes amongst the genes from (**a**), as a percentage of all genes specific to that cell class. (**c**) Statistically significantly enriched cell classes amongst the mouse genes expressed rhythmically in the eye under LD conditions which were assigned specific retinal cell classes, as a percentage of all genes specific to that cell class. (**b**,**c**) Enrichment analysis carried out by one-tailed Fisher’s exact test (** *p* < 0.01, *** *p* < 0.001, **** *p* < 0.0001). (**d**) Overlap between rhythmic genes in the mouse eye in LD and DD conditions which are rod-, cone- and photoreceptor-specific.

**Figure 5:**
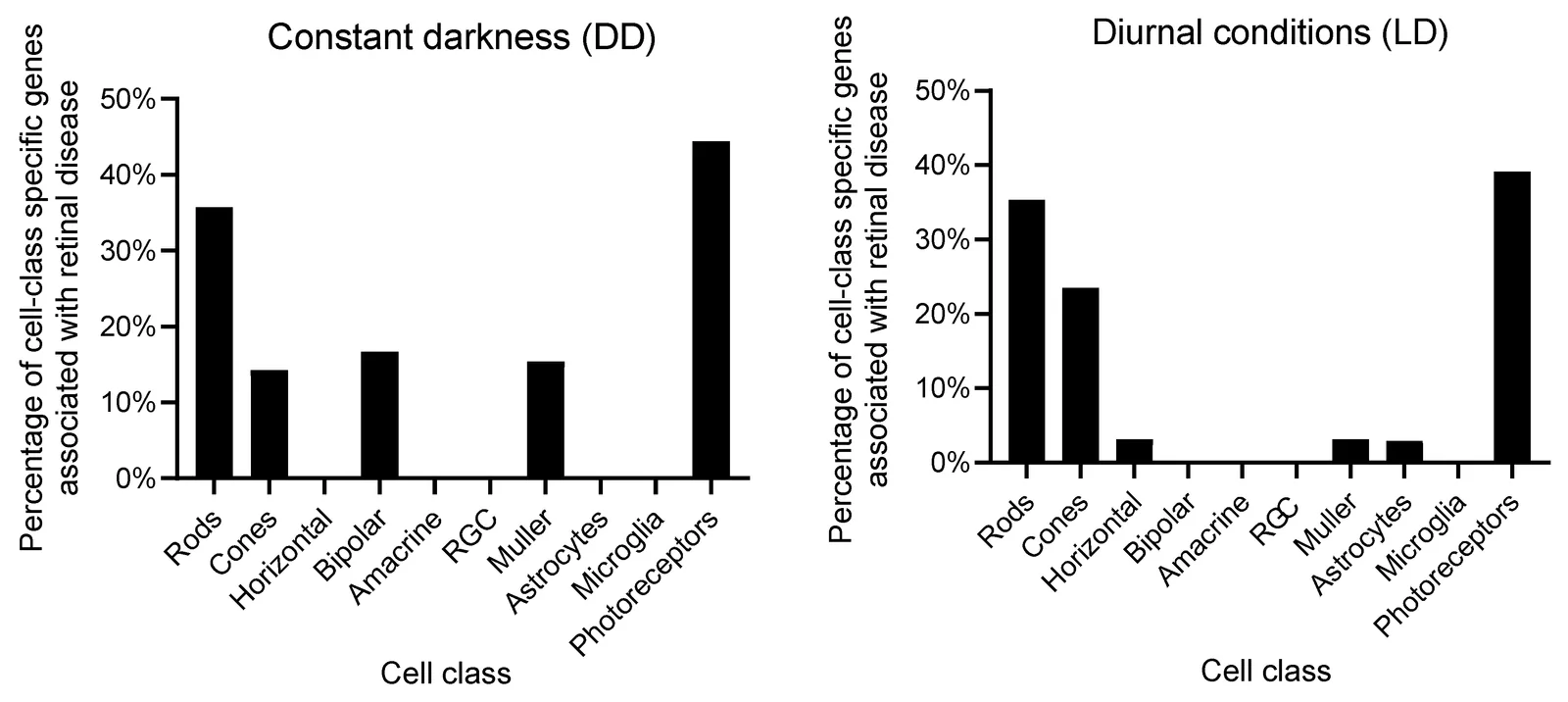
Percentage of circadian mouse retinal genes specific to each cell class associated with monogenic retinopathy.

**Table 1:** Circadian photoreceptor-specific genes which may contribute to outer segment shedding.

Gene	Protein	Cell Class	Role in Outer Segment Shedding
*Pcare*	PCARE	Photoreceptors	Localises to connecting cilia [28]. Associates with Arp2/3 complex activator Wasf3 to drive ciliary tip expansion for outer disc formation [29,30,31]. Essential for photoreceptor survival [32,33]; mutations cause autosomal recessive retinitis pigmentosa [34].
*Agpat3*	1-acylglycerol-3-phosphate O-acyltransferase 3	Photoreceptors	Encodes a lysophosphatidic acid acyltransferase. May contribute to rhythmic membrane lipid biosynthesis for outer disc formation.
*Pde4dtp*	Myomegalin	Rods	Interacts with Fam161a and the USH complex at the base of the connecting cilia [35,36,37]. May contribute to a scaffolding complex allowing dynein-mediated transport to the outer segment.
*Fscn2*	Fascin-2 (retinal fascin)	Rods	Cross links and stabilises actin in outer segments [38]. Mutations cause autosomal dominant retinitis pigmentosa and/or macular degeneration [39,40,41].
*Kif2a*	KIF2A	Cones	Promotes neuronal microtubule depolymerisation [42,43]. Specific retinal role not investigated, but may have a role in cilial function.

## Data Availability

The data analysed in this study is derived from the following open-access publications, as cited in the main body of the paper: ref. [22] Lukowski, S.W. et al., A single-cell transcriptome atlas of the adult human retina. *EMBO J*, **2019**. *38*(18): p. e100811, doi:10.15252/embj.2018100811. ref. [11] Storch, K.F. et al., Intrinsic circadian clock of the mammalian retina: importance for retinal processing of visual information. *Cell*, **2007**. *130*(4): p. 730–741, doi:10.1016/j.cell.2007.06.045. The original contributions presented in this study are included in the article and Supplementary Materials. Further inquiries can be directed to the corresponding authors.

## Supplementary Materials

The following supporting information can be downloaded at https://www.mdpi.com/article/10.3390/clockssleep8040060/s1. Supplementary Data S1: Retinal Cell Mapping model (Microsoft Excel), the development and validation of which was described within this paper. This model assigns retinal cell-class specificity to genes if applicable and assesses for statistical enrichment of any particular retinal cell-classes in gene lists. Supplementary Data S2: Complete list of genes assigned a specific cell class by the Retinal Mapping Model. This includes human genes, and mouse genes with 1:1 orthology. Supplementary Data S3: Supplementary figures and tables. Supplementary Data S4: Raw data for Figure 2, Figure 3, Figure 4 and Figure 5, Supplementary Figures S1–S3. Supplementary Figure S1: The ‘Others’ clusters express markers of multiple retinal cell types. Supplementary Figure S2: Human retinal expression patterns of the nine in vivo verified retinal cell class marker genes which failed to be assigned the correct cell class by the validated retinal cell mapping model. Supplementary Figure S3: Validation of parameters for the ‘photoreceptors’ category. Supplementary Table S1: Genes displaying rhythmicity under DD (constant darkness) conditions in the mouse eye to which the model could be applied, and the cell classes to which they were assigned if applicable. Supplementary Table S2: Genes displaying rhythmicity under LD (12 h light-dark cycle) to which the model could be applied, and the cell classes to which they were assigned if applicable. Supplementary Table S3: Cell class-specific genes with circadian expression in the mouse eye in diurnal conditions (LD) which are associated with human retinopathies. Supplementary Discussion S5: Exploration of the potential biological relevance of the rhythmic genes identified as astrocyte-specific in our model. Table S4: The role of glucocorticoid-regulated astrocyte-specific genes in retinal astrocytes.

## Abbreviations

The following abbreviations are used in this manuscript:

DD	Constant darkness (“dark-dark”)
ERG	Electroretinogram
ipRGC	Intrinsically photosensitive retinal ganglion cell
LD	Light-dark
RGC	Retinal ganglion cell
RNA-seq	RNA sequencing
RPE	Retinal pigment epithelium
scRNA-seq	Single-cell RNA sequencing
TTFL	Transcriptional-translational feedback loop

## Appendix A

**Table A1:** Cell class specific markers in the retina, and in vivo evidence for their specificity as identified in the literature, including the mammalian species in which this evidence was obtained.

Cell Class	Marker Gene	References	Species
Rods	*CNGA1*	[81,82,83]	Mouse, rat, cat
	*CNGB1*	[84,85]	Human, cow
	*GNAT1*	[86,87]	Cow, human
	*NR2E3*	[88]	Macaque, mouse
	*NRL*	[89]	Human
	*PDE6A*	[90,91,92]	Cow, mouse
	*PDE6B*	[91,92,93,94]	Cow, mouse, dog
	*PDE6G*	[91,93,94,95,96]	Cow, mouse, dog, ferret
	*RHO*	[97,98,99,100]	Cow, mouse, tree shrew
	*ROM1*	[98,101,102,103]	Cow, human, mouse
	*SAG*	[93,104,105,106]	Human, cat, cow, dog
	*SAMD11*	[107,108]	Mouse
	*SAMD7*	[109]	Mouse
	*SLC24A1*	[110,111]	Cow
Cones	*ARR3*	[97,105,112,113,114]	Rat, human, cat, cow, macaque, mouse
	*CNGA3*	[82,115,116]	Mouse, cow
	*CNGB3*	[81,82]	Mouse
	*GNAT2*	[87,97,117]	Cow, mouse, human
	*GNGT2*	[117,118]	Human, cow, mouse
	*OPN1LW*	[86,119,120,121]	Human, macaque
	*OPN1MW*	[97,120,121,122]	Mouse, macaque
	*OPN1SW*	[82,97,121,122]	Mouse
	*PDE6C*	[91,92,123,124,125]	Cow, mouse
	*PDE6H*	[91,95,126,127]	Cow, mouse, rat
	*SLC24A2*	[128]	Mouse
	*THRB*	[129,130]	Mouse
Horizontal cells	*LHX1*	[131]	Mouse
	*ONECUT1*	[132]	Mouse
	*ONECUT2*	[132]	Mouse
	*TMOD1*	[133]	Mouse
Bipolar cells	*CA10*	[134]	Mouse
	*CA8*	[134,135]	Mouse, rat, macaque
	*CABP5*	[136]	Mouse, cow, baboon, human
	*GNAO1*	[137]	Cat, monkey, rat, cow
	*GRM6*	[138,139]	Monkey, rat
	*GSG1*	[140]	Mouse
	*LHX4*	[141]	Mouse
	*PCP2*	[142]	Mouse, rabbit
	*PRKCA*	[83,143]	Cat, rat, rabbit, macaque
	*TMEM215*	[140]	Mouse
	*TRNP1*	[140]	Mouse
	*TRPM1*	[134,144]	Human, mouse
	*VSX1*	[145]	Mouse
	*VSX2*	[146]	Mouse
Amacrine cells	*CHAT*	[147]	Rat
	*GABRD*	[143]	Rat
	*GAD1*	[148,149,150]	Rat, rabbit, human, monkey
	*LHX9*	[141]	Mouse
	*SLC18A3*	[151]	Rat
	*SLC6A1*	[64,152]	Macaque, human, rat
	*SLC6A9*	[65,66,67]	Cat, rat, rabbit, chicken, macaque, human
	*STX1A*	[153]	Rat
Retinal ganglion cells	*MAP1A*	[154]	Rat
	*NEFH*	[155,156]	Rat, pig
	*NEFL*	[155]	Human, pig
	*NEFM*	[155]	Human, pig
	*POU4F1*	[157]	Rat
	*POU4F2*	[158]	Chicken, mouse, rabbit, monkey, human
	*RBPMS*	[159,160,161]	Rat, mouse, guinea pig, rabbit, monkey, marmoset, human
	*SLC6A17*	[162]	Cow
	*SNCG*	[163,164]	Human, mouse
	*THY1*	[165,166,167]	Rat, mouse
	*TUBB3*	[168,169]	Mouse, rat
Muller cells	*CA2*	[70]	Mouse
	*GLUL*	[68,69,70,71]	Rat, mouse, cat
	*RDH10*	[170]	Cow
	*RGR*	[171]	Cow
	*RLBP1*	[172]	Cow, monkey
	*SLC6A11*	[173]	Rat
Astrocytes	*GFAP*	[174,175]	Human, rat
	*NDRG2*	[176]	Marmoset
	*PAX2*	[177]	Guinea pig, mouse, dog, monkey
	*PAX8*	[178]	Mouse
Microglia	*AIF1*	[179]	Mouse
	*C1QA*	[180]	Mouse
	*CD68*	[179]	Mouse
	*CD74*	[181]	Rat
	*HLA-DPA1*	[182,183]	Rat, pig
	*HLA-DQA1*	[182,183]	Rat, pig
	*HLA-DRA*	[182,183]	Rat, pig
	*ITGAM*	[184]	Mouse
Photoreceptors	*CRX*	[185]	Monkey, mouse
	*ELOVL4*	[186]	Human, mouse, cow, cat, rat
	*FSCN2*	[187]	Rat
	*GRK1*	[188]	Human
	*GUCY2D*	[189]	Rhesus monkey
	*IMPG1*	[190]	Human
	*PDC*	[191,192]	Mouse, human
	*RBP3*	[193]	Human, ground squirrel
	*RCVRN*	[194]	Mouse, cow
	*SLC4A7*	[195,196]	Mouse
	*TULP1*	[197]	Mouse
Retinal pigment epithelium	*BEST1*	[198]	Pig, macaque
	*DCT*	[199]	Mouse
	*PDPN*	[200]	Mouse
	*RPE65*	[201]	Cow
	*RRH*	[202]	Mouse
	*SLC16A8*	[203]	Rat
	*TYRP1*	[204]	Mouse
