# Supplementary material for: Mapping Retinal Gene Expression to Cell Classes: Insights into Retinal Circadian Rhythms
Source: Clocks Sleep. 2026 Sep 25;8(4):60. doi: 10.3390/clockssleep8040060 (PMC13692337; doi:10.3390/clockssleep8040060)
Supplement: Supplementary file 1 [file clockssleep-08-00060-s001.zip › SD3 Supplementary Figures and Tables.pdf]

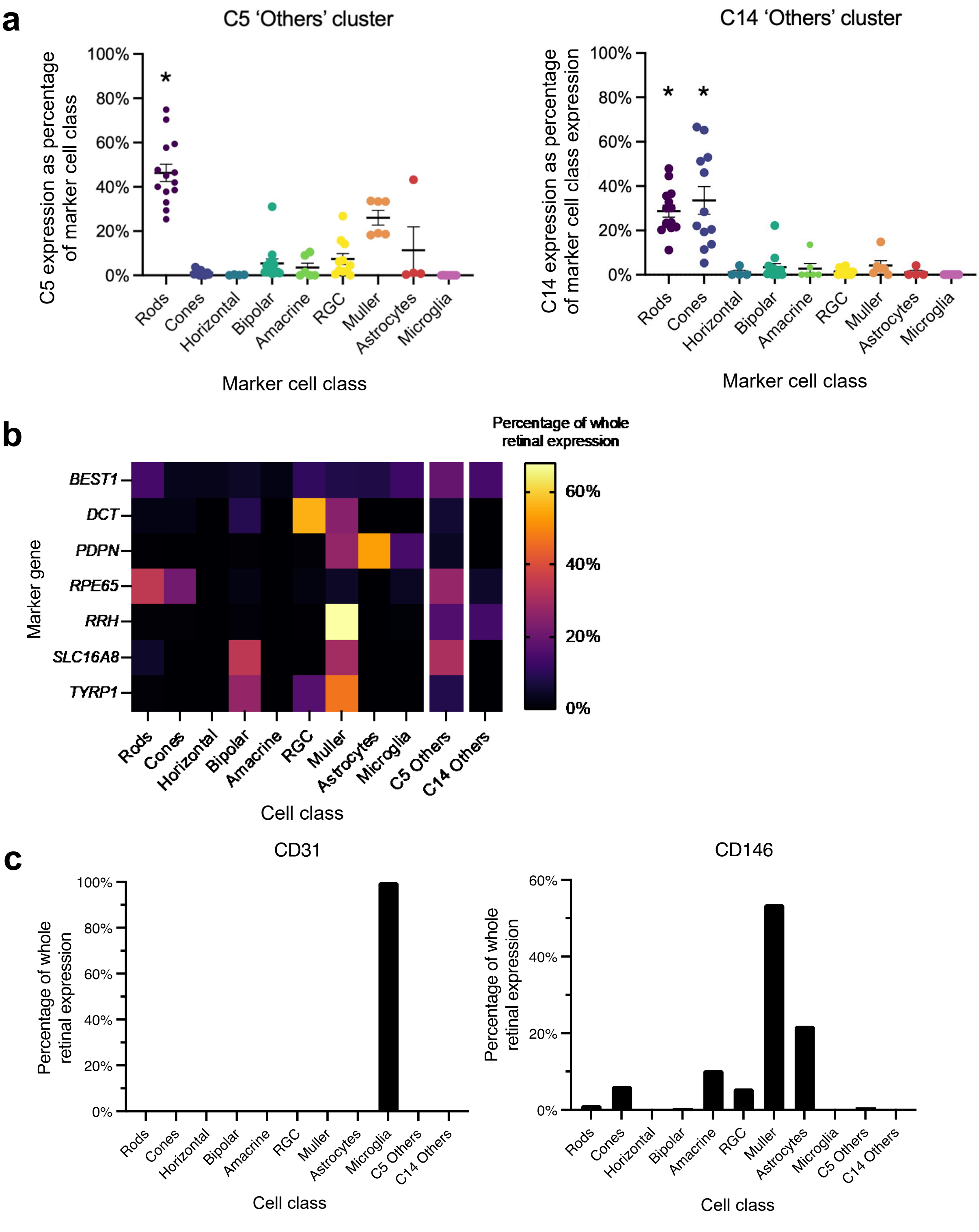

Supplementary Figure S1 The 'Others' clusters express markers of multiple retinal cell classes. (**a**) Expression of each cell class' marker genes in the C5 or C14 'Others' cluster as a percentage of expression in the marker cell class. Each data point represents one marker gene. Statistical comparison made using Kruskal-Wallis analysis ($p<0.0001$ for both). Multiple comparisons testing carried out using Dunn's test; cell classes wherein percentage expression of markers in the indicated 'Others' cluster is significantly higher ($p<0.05$) than at least five other cell classes are

indicated with a *. Bars represent standard error of the mean. (**b**) Percentage of expression of RPE marker genes in each retinal cell class. (**c**) Percentage of expression of the endothelial markers CD31 (*PECAM1*) and CD146 (*MCAM*) in each retinal cell class. All expression values are from the source scRNA-seq dataset (Lukowski et al. 2019).

Bipolar cell markers

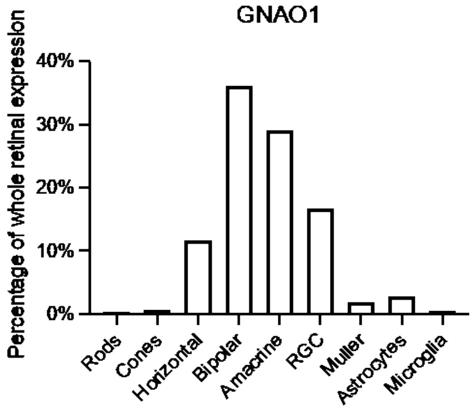

Cell class **with maximum expression:** Bipolar
Cell class **assigned by model:** None

Amacrine cell markers

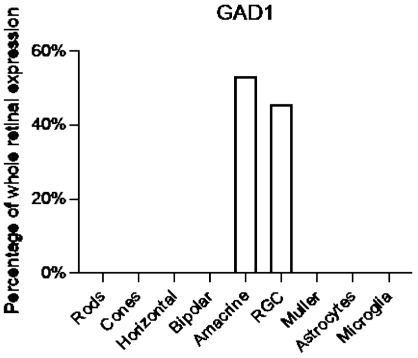

Cell class **with maximum expression:** Amacrine
Cell class **assigned by model:** None

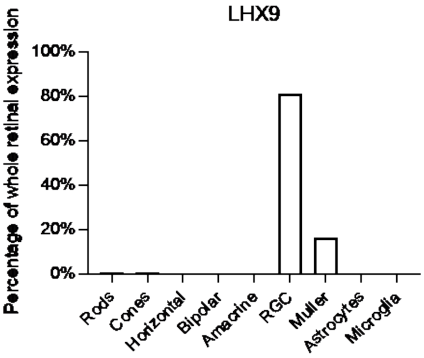

Cell class **with maximum expression:** RGC
Cell class **assigned by model:** RGC

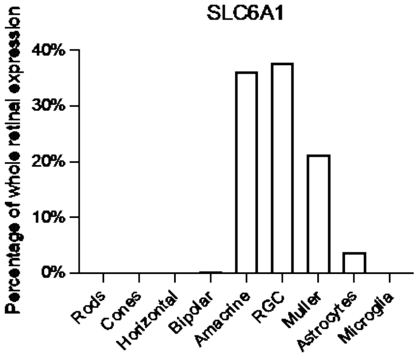

Cell class **with maximum expression:** RGC
Cell class **assigned by model:** None

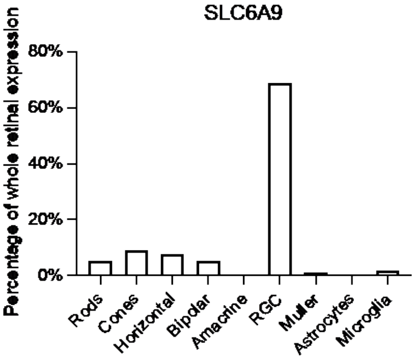

Cell class **with maximum expression:** RGC
Cell class **assigned by model:** RGC

RGC markers

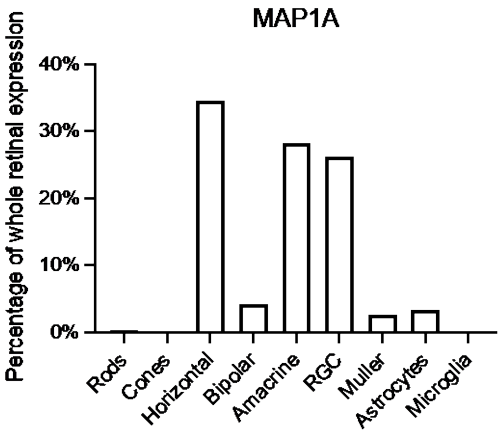

Cell class **with maximum expression:** Horizontal
Cell class **assigned by model:** None

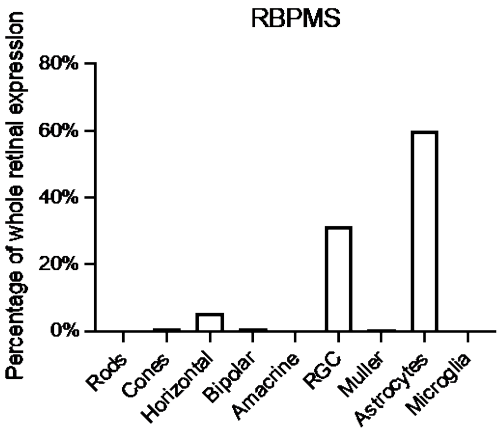

Cell class **with maximum expression:** Astrocytes
Cell class **assigned by model:** Astrocytes

Müller cell markers

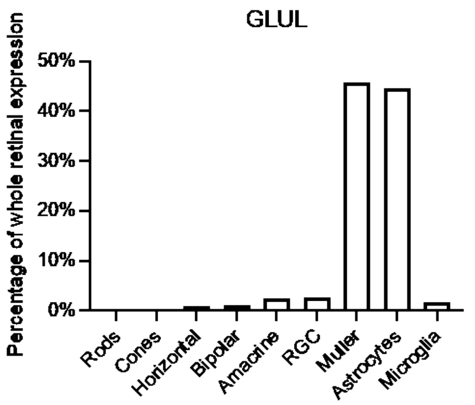

Cell class **with maximum expression:** Müller cells
Cell class **assigned by model:** None

Astrocyte markers

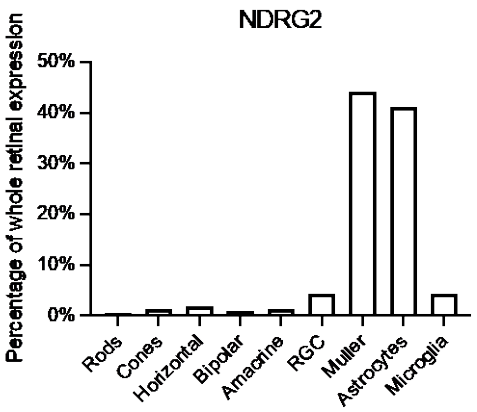

Cell class **with maximum expression:** Müller cells
Cell class **assigned by model:** None

Supplementary Figure S2 Human retinal expression patterns of the nine *in vivo* verified retinal cell class marker genes which failed to be assigned the correct cell class by the validated retinal cell mapping model. All expression values are from the source scRNA-seq dataset (Lukowski et al. 2019).

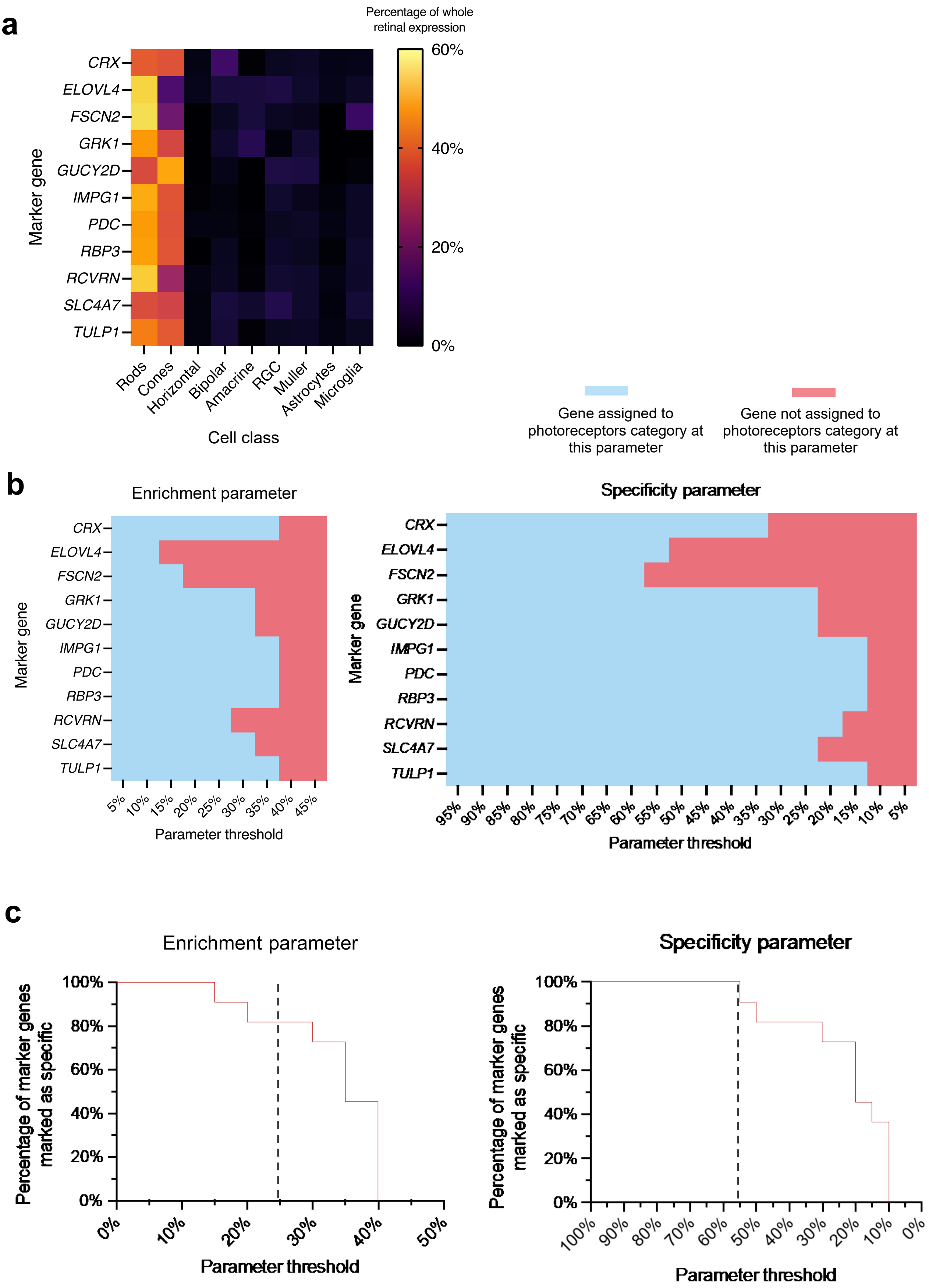

Supplementary Figure S3 Validation of parameters for the 'photoreceptors' category. (**a**) Heatmap showing percentage expression in each retinal cell class of the identified photoreceptor-specific

marker genes. (**b**) Parameter values for the expression and specificity parameters at which each photoreceptor marker gene is no longer assigned to the photoreceptors category. (**c**) Percentage of photoreceptor-specific genes assigned to the photoreceptors category at each parameter value for both the expression parameter and specificity parameter. (**b, c**) Finalised parameter values are indicated with a dotted line.

Supplementary Table S1. Genes displaying rhythmicity under DD (constant darkness) conditions in the mouse eye to which the model could be applied, and the cell classes to which they were assigned if applicable. Data from Storch et al. 2007.

**Rods**	*Fabp12, Fmo1, Fscn2, Glb1l2, Hsp90aa1, Nr2e3, Pde4dip, Pde6b, Pde6g, Plekhf2, Rcvrn, Rims2, Sag, Usp2*
**Cones**	*Adcy1, Ankrd33b, Aqp1, Arr3, Best2, Frmpd1, Frmpd2, Guca1b, H2aw, Kif2a, Rgs20, Rs1, Slc25a20, Slc4a11*
**Horizontal**	*Hdac9, Nfil3, Sqle, Tcap, Trim25*
**Bipolar**	*Car8, Fgf12, Khdrbs3, Lhx3, Pdzrn3, Trpm1*
**Amacrine**	*Mab21l2, Memo1, Slc7a1, Snap25, Thra, Urb1*
**RGC**	*Ctif, Fam102b, Fut9, Spon2, Sptssb, Thbs1*
**Müller**	*Bhlhe40, Car14, Car3, Ccn2, Col4a6, Crispld2, Cys1, Efnb2, Fech, Lrat, Mmp14, Spc25, Timp3*
**Astrocytes**	*Angptl7, Cd44, Cdkn1a, Elk3, Epas1, Fmo2, Por, Rhoj, Slc4a4, Tgm2, Tmem229b, Ttll11*
**Microglia**	*Acsl5, Actb, Adrb2, Def6, Il18, Myl12a, Prf1, Tsc22d3*
**Photoreceptors**	*Agpat3, Cldn7, Crx, Grtp1, Guca1a, Hcn1, Pcare, Pcdh15, Plekhb1*
**Not assigned specific cell class**	*Acadm, Actg1, Adk, Ak4, Alkbh3, Arl3, Asprv1, Atp1b3, Aurkc, Bbs10, Btbd9, C1qbp, C2cd2l, Cbfa2t2, Ccnd1, Cerk, Ciao1, Cirbp, Cks2, Cnot6, Cstb, Dbp, Dhcr24, Dleu7, Dnajb2, Drd4, Dusp1, Egln3, Eno2, Ero1a, Fam3c, Fus, Gars, Glmn, Gnaz, H1f0, H3f3b, Hmgb2, Hmgcs1, Hnrnpd, Hnrnpdl, Hnrnpk, Hsp90b1, Hspa8, Idi1, Ip6k2, Irf7, Josd2, Kank1, Ltv1, Mast2, Med13l, Mef2d, Mgat4a, Mgll, Mknk2, Mov10l1, Mrps2, Msi2, Msmo1, Mxi1, Naa15, Ndrg3, Nfya, Nr1d1, Nr1d2, Nt5c2, Odc1, Opa1, Otud7b, Pbx2, Pcmtd2, Pdap1, Pde8b, Pdzd8, Pea15a, Per2, Per3, Pgk1, Phgdh, Pitpnm3, Pnpla3, Ppp2r2b, Prdm2, Ptgis, Pxmp2, Qrsl1, Sfxn2, Sik2, Smug1, Stard7, Stxbp1, Sv2b, Taf1d, Tef, Tgfb2, Tmem50b, Tpi1, Trpc1, Tspan4, Tsr1, Tubb5, Uri1, Usp4, Vps13a, Washc5, Wdr1, Wdr17, Xrn2, Ywhag, Zbtb18, Zbtb22, Zdhhc5*

Supplementary Table S2. Genes displaying rhythmicity under LD (12-hour light-dark cycle) to which the model could be applied, and the cell classes to which they were assigned if applicable. Genes which were also rhythmic under DD (constant darkness) conditions are shown in bold; see Supplementary Table 1 for complete list. Data from Storch et al. 2007.

**Rods**	*2610528A11Rik, Add1, Aipl1, Arl4d, Cabp4, Cdt1, Cngb1, Dmd,* ***Fabp12****,* ***Fmo1****,* ***Fscn2****,* ***Glb1l2****, Gngt1, Hmgn1,* ***Hsp90aa1****, Kcnb1,* ***Nr2e3****,* ***Pde4dip****,* ***Pde6b****,* ***Pde6g****, Pfkfb2,* ***Plekhf2****, Plpp2, Ppef2,* ***Rcvrn****, Rho,* ***Rims2****, Rpe65,* ***Sag****, Slc24a1, Slco4a1, Ube2c,* ***Usp2****, Ybx3*
**Cones**	*Abcg4, Acsl6,* ***Adcy1****,* ***Ankrd33b****,* ***Aqp1****,* ***Arr3****, Bace1, Cacna2d4, Cdhr1, Clcn4, Cldn5, Cplx4, Ehhadh, Fank1,* ***Frmpd1****,* ***Frmpd2****, Gnat2, Gnb3, Got2,* ***Guca1b****, Guk1,* ***H2aw****, Hk2, Hs3st3b1, Kif21b,* ***Kif2a****, Mef2c, Nol4l, Nxnl2, Opn1sw, Pde6c, Pdzd2, Ppp1r42, Rasgrf2,* ***Rgs20****, Rragd,* ***Rs1****,* ***Slc4a11****, Tctn2, Tfrc*
**Horizontal**	*Ahnak2, Ank2, Ankrd29, Aplp1, Bfsp1, Cdc42ep3, Cxxc5, Cyp51, D430041D05Rik, Dhtkd1, Dyrk2, Fam107b,* ***Hdac9****, Hk1, Ldah, Lurap1l, Mpp3,* ***Nfil3****, Pde8a, Pim1, Prkar1b, Rgs7, Rorb, Rps6kb1, Rttn, Septin4, Slc5a3, Sostdc1,* ***Sqle****,* ***Tcap****,* ***Trim25****, Usp24*
**Bipolar**	*Cabp5, Cacna2d3,* ***Car8****, Ccdc136, Col19a1, Crybg3, Dusp4, Fam171b, Gng13, Gucy1a1,* ***Khdrbs3****, Lmod2, Lrrtm4, Macrod2, Pcp2, Prkca, Prss23, Slc25a22, Trnp1, Ttyh2, Zfp385b*
**Amacrine**	*Arhgef3, Armh4, Asb2, Atp2b2, Cplx2, Dapk1, Dixdc1, Fam89b, Fbn1, Frmd8, Glb1l3, Ikzf4, Mal2, Mcm6,* ***Memo1****, Ppme1, Rnasel, Rrm2, Scg5, Sec14l1, Spen, Taf4b, Tgfb3,* ***Thra****, Zbtb16*
**RGC**	*Anln, Coro2b, Fam102b, Fndc4, Fzr1, Gapdhs, Gpr22, Hmgcr, Kcnab2, Krt19, Npy, Papss2, Pde10a, Rasgrf1, Slc24a3,* ***Spon2****, Synpo2,* ***Thbs1****, Tppp3, Tubb3, Tunar, Vps37d*
**Müller**	*Akap12, Aldh3a1, C130071C03Rik,* ***Car3****, Ccn1,* ***Ccn2****, Cgn, Chrdl1, Col1a1,* ***Crispld2****, Crygn, Csrp1,* ***Cys1****, Dbi, Ehd4, Inhba, Kdr,* ***Lrat****, Macir,* ***Mmp14****, Pde5a, Pdgfra, Pla2r1, Ppp1r3c, Slc25a33,* ***Spc25****, Spon1, Strip2, Tmem150c, Tmem158, Tns4, Vegfd*
**Astrocytes**	*Adora2b, Afap1l2, Amer2,* ***Angptl7****, Antxr2, Aqp4, Arhgef17, Bbs2, Bdh2, Bgn, Casp7, Ccnl2, Clu, Cthrc1, Dad1, Dnpep, Emcn, Erbb3, Fam168a, Gmppa, Gxylt1, Impdh2, Itgb8, Klhl21, Ldlr, Med16, Mre11a, Mrps18a, Osbpl3, Plat, Prkg1, Ptprz1, Pvt1, Rerg, Rgl2,* ***Rhoj****, Sfi1, Slc20a2, Slc35a4, Snx15, Sorcs1, Spice1, Tcn2,* ***Tgm2****, Tmbim1,* ***Tmem229b****, Vasn, Wsb1, Wwp2*
**Microglia**	***Actb****, Adrb2, Arpc2, B2m, C3ar1, Cebpd, Gnai2, H1f2, Ier3,* ***Il18****, Jun, Lhfpl2, Lrmda, Myo1g, Pla2g7, Plac8, Ptafr, Rgs2, Rps6, Sgk1, Tgfbi, Thbd, Tnfsf13,* ***Tsc22d3***
**Photoreceptors**	***Agpat3****, Casz1, Cerkl,* ***Crx****, Fbxo16,* ***Grtp1****,* ***Guca1a****,* ***Hcn1****, Impg1, Lca5, Llgl2, Mak, Paqr4,* ***Pcare****,* ***Pcdh15****, Pdc,* ***Plekhb1****, Sgip1, Slc6a6, Tlcd3b, Unc119, Vtn, Wdr31*
**Not assigned specific cell class**	*2410002F23Rik, 4833439L19Rik, Aacs, Aars, Abhd14a, Abhd2, Abhd3,* ***Acadm****, Acbd4, Acbd6,* ***Actg1****, Actn4, Acyp2, Adipor1, Aff4, Agpat3, AI661453, Alcam, Aldoc,* ***Alkbh3****, Alkbh5, Amotl1, Ankrd39, Anxa1, Ap2a2, Ap5m1, Apbb1, Apc2, Apex1, Araf, Arhgef26, Arih2,* ***Arl3****, Arntl, Aspscr1, Astn1, Atg14, Atp1b2, Atp2a2, Atp6v1a, Atr, Basp1, Bcap29, Bicd2, Bin1, Bnip3, Bnip3l, Brca1, Brd2, Btbd1, Btbd10,* ***C2cd2l****, Cadm1, Calr, Camk2d, Camk4, Casz1, Ccdc173, Ccdc71,* ***Ccnd1****, Cdk2ap2, Cdkn2b, Cds1,* ***Cerk****, Cerkl, Ces4a, Chchd4, Ciart, Cibar1,* ***Cirbp****, Ckb,* ***Cks2****, Col18a1, Col4a1, Col4a4, Coq10a, Cpne3, Cpt1a, Cpt2, Crip1, Crx, Cry1, Csnk2b, Ctbp1, Ctc1, Cystm1, Czib, Dap3,* ***Dbp****, Dcaf8, Dctn1, Ddit4, Ddrgk1, Ddx39a, Dennd10, Dennd5a,* ***Dhcr24****, Dhrs1, Dhrs3, Dipk2a, Dmac1, Dnai4, Dnajb13,* ***Dnajb2****, Dnajb5, Dnajc5, Dnajc9, Dohh, Dpf3, Dph5,* ***Drd4****, Dscaml1,* ***Dusp1****,*

	Echs1, Egln1, ***Egln3****, Ehmt2,* ***Eno2****, Epb41, Epb41l3, Eprs, Eri2, Erlec1,* ***Ero1a****, Fabp7, Fam162a,* ***Fam3c****, Fam53c, Fbxo16, Fbxo45, Fdft1, Fdps, Fgf11, Fn3k, Foxp1,* ***Fus****, Gabarapl1, Gapdh,* ***Gars****, Gas7, Ggt6, Ghr, Gigyf2,* ***Glmn****, Glrx2, Glul,* ***Gnaz****, Gnb5, Got1, Gpsm1, Gpx4, Grtp1, Gtdc1, Guca1a, Gucd1, Gucy1a2, Gyg,* ***H1f0****, H2ax, H2az2,* ***H3f3b****, Hadh, Hbegf, Hbp1, Hcn1, Higd1a, Hipk2, Hmg20b,* ***Hmgb2****,* ***Hmgcs1****,* ***Hnrnpdl****,* ***Hnrnpk****, Hopx,* ***Hsp90b1****, Hspa4l,* ***Hspa8****, Icmt,* ***Idi1****, Ift122, Ift43, Ift81, Ikbkg, Ilrun, Impg1, Ing1,* ***Ip6k2****, Ipmk,* ***Irf7****, Isl1, Itpr1, Jade1, Jchain, Jmjd6, Jmy, Jund, Kcmf1, Kdm4c, Kdm6b, Kif1b, Kif22, Kit, Klc4, Kmt2c, Kmt2e, Kpna2, Krt14, Lca5, Lclat1, Ldha, Lgmn, Lix1l, Llgl2, Lmbr1l, Loxl1, Lpin1, Lrpap1, Lss, Mab21l1, Mak, Mapk14, Marchf5, Marcksl1,* ***Mast2****, Mbnl2, Mcm5, Mcm7, Mdh1, Meak7,* ***Med13l****, Med20,* ***Mef2d****,* ***Mgll****, Mgp, Mgrn1, Mia3, Mif, Mindy2,* ***Mknk2****, Mkrn1, Mmd, Mmd2, Mpdu1,* ***Msmo1****, Mtmr4, Mtmr9,* ***Mxi1****, Mycbp, Myrip, Napb, Ncoa1,* ***Ndrg3****, Nectin1, Nlgn2, Nol6, Nop10, Npepps,* ***Nr1d1****,* ***Nr1d2****, Nrxn3, Nupr1, Oat, Oaz1, Ociad1,* ***Odc1****, Olfm1, Optn, Ormdl3, Oxnad1, P4ha1, Pabpn1, Pank4, Paqr4, Pbrm1, Pcare, Pcbp4, Pcdh15, Pcdh9,* ***Pcmtd2****, Pdc,* ***Pde8b****, Pdia5, Pdk1,* ***Pdzd8****,* ***Pea15a****, Peg3,* ***Per2****,* ***Per3****, Pfdn5, Pfkl, Pfkp,* ***Pgk1****, Phf20, Phf20l1,* ***Phgdh****, Pias3, Pigz, Pik3ca, Pitpnm1,* ***Pitpnm3****, Pkm, Plcd3, Plcxd2, Plekhb1, Plekhb2,* ***Pnpla3****, Pnrc1, Polr3k, Ppa1, Pphln1, Ppp1cc,* ***Ppp2r2b****, Ppp3cb,* ***Prdm2****, Prelid1, Prkab1, Prkcz, Prnp, Prr13, Pspc1, Ptov1, Ptp4a2, Ptpn2, Purb,* ***Pxmp2****,* ***Qrsl1****, Rab24, Rab2a, Rab6a, Rab6b, Racgap1, Rad1, Rad51ap1, Rapgefl1, Rbm3, Rbm6, Rcan2, Rchy1, Rfx1, Rhbdl2, Rian, Ric8b, Rmdn3, Rnf11, Rnf145, Rnf150, Rnf181, Rora, Rprd2, Rps6ka2, Rrp1b, Rwdd4a, Sap30l, Sar1a, Sbk1, Scamp5, Scarb1, Sdccag8, Sdha, Selenon, Serbp1, Serinc3,* ***Sfxn2****, Sgip1, Sgtb, Sh3pxd2a,* ***Sik2****, Sik3, Sirt5, Slc16a3, Slc31a2, Slc6a5, Slc6a6, Smg5, Sms,* ***Smug1****, Smyd5, Snrk, Sod1, Sorl1, Sox12, Spry2, Ssu72, Stard10, Stard4,* ***Stard7****, Stk16, Stt3b, Stx3,* ***Stxbp1****, Susd6, Suv39h2,* ***Sv2b****, Syf2, Syncrip, Syngr1, Synj1, Tagln, Tapbp, Tbrg1, Tcta,* ***Tef****, Tex2, Thap7, Tlcd3b, Tm9sf2, Tmem230, Tmem256, Tmem59l, Tmem63b, Tmem9b, Tnfaip1, Top1,* ***Tpi1****, Tpm1, Tprkb,* ***Trpc1****,* ***Tspan4****, Tubb4b, Txndc16, Ube3b, Ubxn2a, Uckl1, Ulk1, Unc119,* ***Uri1****, Usp19, Vamp2, Vdac1, Vps25, Vtn, Wdr17, Wdr31, Wdr5, Wdr70, Wee1, Wrnip1, Xab2, Xbp1, Xpo1, Ypel2, Zbtb1, Zbtb20,* ***Zdhhc5****, Zfc3h1, Zfp292, Zfp329, Zfp410, Zfp467, Zfp771, Zfp90, Zfyve28, Zmym2, Zmym3, Zmynd8*

Supplementary Table S3. Cell class-specific genes with circadian expression in the mouse eye in diurnal conditions (LD) which are associated with human retinopathies. Genes also rhythmic in the mouse eye in constant darkness (DD) are shown in bold. Bipolar cells, amacrine cells, RGCs and microglia are omitted as no diurnal circadian genes specific to these cell classes were associated with disease. Disease-associated, cell class-specific rhythmic in DD but not LD are described below. Data from Storch et al. 2007. Retinal disease-associated genes obtained from RetNet (www.retnet.org).

	**Rods**	**Cones**	**Horizontal**	**Muller**	**Astrocytes**	**Photoreceptors**
**Bardet-Biedl syndrome**, autosomal recessive					*Bbs2*	
**Cone or cone-rod dystrophy**, autosomal dominant	*Aipl1*					***Crx***, ***Guca1a***, *Unc119*
**Cone or cone-rod dystrophy**, autosomal recessive		*Cacna2d4*, *Cdhr1*, *Gnat2*, *Pde6c*				*Cerkl*
**Congenital stationary night blindness**, autosomal dominant	***Pde6b***, *Rho*					
**Congenital stationary night blindness**, autosomal recessive	*Cabp4*, ***Sag***, *Slc24a1*	*Gnb3*				
**Deafness alone or syndromic**, autosomal recessive						***Pcdh15***
**Leber congenital amaurosis**, autosomal dominant						***Crx***
**Leber congenital amaurosis**, autosomal recessive	*Aipl1*, *Cabp4*, *Rpe65*			***Lrat***		***Crx***, *Lca5*
**Macular degeneration**, autosomal dominant	***Fscn2***	***Guca1b***				*Impg1*
**Macular degeneration**, autosomal recessive						*Impg1*
**Retinitis pigmentosa**, autosomal dominant	***Fscn2***, ***Nr2e3***, *Rho*, *Rpe65*, ***Sag***	***Guca1b***	*Hk1*			***Crx***, *Impg1*
**Retinitis pigmentosa**, autosomal recessive	*Cngb1*, ***Nr2e3***, ***Pde6b***, ***Pde6g***, *Rho*, *Rpe65*, ***Sag***			***Lrat***	*Bbs2*	*Cerkl*, *Mak*, ***Pcare***
**Usher syndrome**, autosomal recessive						***Pcdh15***

**Other retinopathy**, autosomal dominant		*Opn1sw*				
**Other retinopathy**, autosomal recessive	***Nr2e3***					
**Other retinopathy**, X-linked	*Dmd*	***Rs1***				

Two cell class specific circadian genes in constant darkness (DD) associated with disease were not rhythmic in diurnal conditions (LD). These were:

- *Trpm1* – Bipolar cells
  - Congenital stationary night blindness, autosomal recessive
- *Timp3* – Müller cells
  - Macular degeneration, autosomal dominant
