# Supplementary material for: Mapping Retinal Gene Expression to Cell Classes: Insights into Retinal Circadian Rhythms
Source: Clocks Sleep. 2026 Sep 25;8(4):60. doi: 10.3390/clockssleep8040060 (PMC13692337; doi:10.3390/clockssleep8040060)
Supplement: Supplementary file 1 [file clockssleep-08-00060-s001.zip › SD5 Supplementary Discussion.pdf]

<u>Supplementary Discussion</u>: *Exploration of the potential biological relevance of the rhythmic genes identified as astrocyte-specific in our model.*

Of the nine astrocyte-specific rhythmic genes in DD conditions, five are regulated by glucocorticoids [1], so their rhythmicity may be contributed to by circadian oscillations in systemic cortisol [2]. These contribute to many astrocyte functions (**Table S4**), such as hypoxia response and neurovascular coupling. However, some functions are contradictory. Though tissue transglutaminase is essential for vascular leakage in diabetic mouse retina [3], HIF-2α stimulates erythropoietin release which is protective in a rat model of diabetic retinal oedema [4, 5]. Nonetheless, glucocorticoids are protective in oedematous retinal diseases [6, 7], so the protective action of pathways such as HIF-2α-EPO dominates. The circadian, cortisol-mediated nature of these pathways indicates that administration of intravitreal glucocorticoids may be more effective later in the day when systemic cortisol decreases [2]. Benefits of similar glucocorticoid chronotherapy have been demonstrated in rheumatoid arthritis [8], so warrant investigation in glucocorticoid-sensitive retinal pathologies such as oedema and non-infectious uveitis [9].

Table S4. The role of glucocorticoid-regulated astrocyte-specific genes in retinal astrocytes.

Gene	Protein	Role in astrocytes and/or glucocorticoid response
Angpt17	Angiopoietin-related protein 7	Not studied in astrocytes or the retina. Induced by dexamethasone in trabecular meshwork cells [10, 11], and remodels extracellular matrix [12]. Missense variants are protective against glaucoma [13]. Induced by dexamethasone in corneal fibroblasts [14]. Antiangiogenic and maintains corneal transparency [15].
Cdkn1a	p21	CDK inhibitor inducing cell cycle arrest in G1. Key transcriptional target of p53. Corticosteroids inhibit astrocyte proliferation [16] and p21 likely a major contributor to this response.
Epas1	Hypoxia-inducible factor 2α (HIF2α)	HIF-2 required for secretion of neuroprotective factors by astrocytes in hypoxia [5]. In oxygen-glucose deprivation, HIF-2 induces EPO secretion from astrocytes but not VEGF or LDH, whereas HIF-1 does the opposite [5]. Erythropoietin is protective in the retina [17], particularly in the context of hypoxia, such as transient retinal ischaemia [18] and diabetic retinal oedema [4].
Por	Cytochrome p450 oxidoreductase	Required for cytochrome p450 function [19]. CYP450 epoxygenase catalyses synthesis of epoxyeicosatrienoic acids [20], which are required for vasodilation upon retinal glial stimulation [21]. CYP450s appear to contribute to retinal astrocytic VEGF release in hypoxia [22].
Tgm2	Tissue transglutaminase	Knockdown improves astrocyte survival in oxygen-glucose deprivation [23, 24]. Activity enhanced by inflammatory cytokines [25]. Tissue transglutaminase activated by VEGF is essential for vascular leakage in diabetic mouse retina [3]. Involved in extracellular matrix regulation as well as

	astrocyte adhesion to fibronectin [25, 26]. May have role in glial scarring.

1. Yan, J., et al., Analysis of gene regulatory networks in the mammalian circadian rhythm. *PLoS Comput Biol*, **2008**. *4*(10): p. e1000193.
2. Bliss, E.L., et al., The normal levels of 17-hydroxycorticosteroids in the peripheral blood of man. *J Clin Invest*, **1953**. *32*(9): p. 818-23.
3. Lee, Y.J., et al., Essential Role of Transglutaminase 2 in Vascular Endothelial Growth Factor-Induced Vascular Leakage in the Retina of Diabetic Mice. *Diabetes*, **2016**. *65*(8): p. 2414-28.
4. Brines, M., et al., Nonerythropoietic, tissue-protective peptides derived from the tertiary structure of erythropoietin. *Proc Natl Acad Sci U S A*, **2008**. *105*(31): p. 10925-30.
5. Chavez, J.C., et al., The transcriptional activator hypoxia inducible factor 2 (HIF-2/EPAS-1) regulates the oxygen-dependent expression of erythropoietin in cortical astrocytes. *J Neurosci*, **2006**. *26*(37): p. 9471-81.
6. Jonas, J.B., I. Kreissig, and R. Degenring, Intravitreal triamcinolone acetonide for treatment of intraocular proliferative, exudative, and neovascular diseases. *Prog Retin Eye Res*, **2005**. *24*(5): p. 587-611.
7. Kim, E.J., et al., Treatment of Diabetic Macular Edema. *Curr Diab Rep*, **2019**. *19*(9): p. 68.
8. Cutolo, M., Glucocorticoids and chronotherapy in rheumatoid arthritis. *RMD Open*, **2016**. *2*(1): p. e000203.
9. Valdes, L.M. and L. Sobrin, Uveitis Therapy: The Corticosteroid Options. *Drugs*, **2020**. *80*(8): p. 765-773.
10. Lo, W.R., et al., Tissue differential microarray analysis of dexamethasone induction reveals potential mechanisms of steroid glaucoma. *Invest Ophthalmol Vis Sci*, **2003**. *44*(2): p. 473-85.
11. Rozsa, F.W., et al., Gene expression profile of human trabecular meshwork cells in response to long-term dexamethasone exposure. *Mol Vis*, **2006**. *12*: p. 125-41.
12. Comes, N., L.K. Buie, and T. Borras, Evidence for a role of angiopoietin-like 7 (ANGPTL7) in extracellular matrix formation of the human trabecular meshwork: implications for glaucoma. *Genes Cells*, **2011**. *16*(2): p. 243-59.
13. Tanigawa, Y., et al., Rare protein-altering variants in ANGPTL7 lower intraocular pressure and protect against glaucoma. *PLoS Genet*, **2020**. *16*(5): p. e1008682.
14. Liu, L., et al., Gene expression and miR profiles of human corneal fibroblasts in response to dexamethasone. *Invest Ophthalmol Vis Sci*, **2011**. *52*(10): p. 7282-8.
15. Toyono, T., et al., Angiopoietin-like 7 is an anti-angiogenic protein required to prevent vascularization of the cornea. *PLoS One*, **2015**. *10*(1): p. e0116838.
16. Crossin, K.L., et al., Glucocorticoid receptor pathways are involved in the inhibition of astrocyte proliferation. *Proc Natl Acad Sci U S A*, **1997**. *94*(6): p. 2687-92.
17. Luo, W., L. Hu, and F. Wang, The protective effect of erythropoietin on the retina. *Ophthalmic Res*, **2015**. *53*(2): p. 74-81.
18. Junk, A.K., et al., Erythropoietin administration protects retinal neurons from acute ischemia-reperfusion injury. *Proc Natl Acad Sci U S A*, **2002**. *99*(16): p. 10659-64.
19. Fluck, C.E., C. Nicolo, and A.V. Pandey, Clinical, structural and functional implications of mutations and polymorphisms in human NADPH P450 oxidoreductase. *Fundam Clin Pharmacol*, **2007**. *21*(4): p. 399-410.
20. Spector, A.A., Arachidonic acid cytochrome P450 epoxygenase pathway. *J Lipid Res*, **2009**. *50 Suppl*(Suppl): p. S52-6.
21. Metea, M.R. and E.A. Newman, Glial cells dilate and constrict blood vessels: a mechanism of neurovascular coupling. *J Neurosci*, **2006**. *26*(11): p. 2862-70.
22. Capozzi, M.E., G.W. McCollum, and J.S. Penn, The role of cytochrome P450 epoxygenases in retinal angiogenesis. *Invest Ophthalmol Vis Sci*, **2014**. *55*(7): p. 4253-60.
23. Colak, G. and G.V. Johnson, Complete transglutaminase 2 ablation results in reduced stroke volumes and astrocytes that exhibit increased survival in response to ischemia. *Neurobiol Dis*, **2012**. *45*(3): p. 1042-50.
24. Feola, J., et al., Transglutaminase 2 modulation of NF-kappaB signaling in astrocytes is independent of its ability to mediate astrocytic viability in ischemic injury. *Brain Res*, **2017**. *1668*: p. 1-11.

25. Espitia Pinzon, N., et al., Tissue transglutaminase in astrocytes is enhanced by inflammatory mediators and is involved in the formation of fibronectin fibril-like structures. *J Neuroinflammation*, **2017**. *14*(1): p. 260.
26. van Strien, M.E., et al., Astrocyte-derived tissue transglutaminase interacts with fibronectin: a role in astrocyte adhesion and migration? *PLoS One*, **2011**. *6*(9): p. e25037.
